# Adjusting FRAX Estimates of Fracture Probability Based on a Positive Vertebral Fracture Assessment

**DOI:** 10.1001/jamanetworkopen.2023.29253

**Published:** 2023-08-17

**Authors:** Carrie Ye, William D. Leslie, Suzanne N. Morin, Lisa M. Lix, Eugene V. McCloskey, Helena Johansson, Nicholas C. Harvey, Mattias Lorentzon, John A. Kanis

**Affiliations:** 1 Department of Medicine, University of Alberta, Edmonton, Alberta, Canada; 2 Department of Medicine, University of Manitoba, Winnipeg, Manitoba, Canada; 3 Department of Medicine, McGill University, Montreal, Quebec, Canada; 4 Centre for Metabolic Bone Diseases, University of Sheffield Medical School, Sheffield, United Kingdom; 5 Medical Research Council (MRC) Versus Arthritis Centre for Integrated Research Into Musculoskeletal Ageing, Department of Oncology and Metabolism, University of Sheffield, Sheffield, United Kingdom; 6 Mary McKillop Institute for Health Research, Australian Catholic University, Melbourne, Victoria, Australia; 7 MRC Lifecourse Epidemiology Centre, University of Southampton, Southampton, United Kingdom; 8 National Institute for Health and Care Research Southampton Biomedical Research Centre, University of Southampton and University Hospital Southampton National Health Service Foundation Trust, Southampton, United Kingdom; 9 Sahlgrenska Osteoporosis Centre, University of Gothenburg, Gothenburg, Sweden; 10 Region Västra Götaland, Sahlgrenska University Hospital, Mölndal, Sweden

## Abstract

**Importance:**

FRAX is the most widely used and validated fracture risk prediction tool worldwide. Vertebral fractures, which are an indicator of subsequent osteoporotic fractures, can be identified using dual-energy x-ray absorptiometry (DXA) vertebral fracture assessment (VFA).

**Objective:**

To assess the calibration of FRAX and develop a simple method for improving FRAX-predicted fracture probability in the presence of VFA-identified fracture.

**Design, Setting, and Participants:**

This prognostic study analyzed the DXA and VFA results of all individuals who underwent a VFA between March 31, 2010, and March 31, 2018, who were included in the Manitoba Bone Mineral Density Registry. These individuals were randomly assigned to either the development cohort or validation cohort. A modified algorithm-based qualitative approach was used by expert readers to code VFAs as positive (≥1 vertebral fractures detected) or negative (0 vertebral fracture detected). Statistical analysis was conducted from August 7, 2022, to May 22, 2023.

**Exposures:**

FRAX scores for major osteoporotic fracture (MOF) and hip fracture were calculated with or without VFA results.

**Main Outcomes and Measures:**

Incident fractures and death were ascertained using linked population-based health care provincial data. Cumulative incidence curves for MOF and hip fracture were constructed, including competing mortality, to predict the 10-year observed risk of fracture. The observed probability was compared with FRAX-predicted fracture probability with and without VFA results and recalibrated FRAX from derived multipliers.

**Results:**

The full cohort of 11 766 individuals was randomly allocated to the development cohort (n = 7854; 7349 females [93.6%]; mean [SD] age, 75.7 [6.8] years) or the validation cohort (n = 3912; 3713 females [94.9%]; mean [SD] age, 75.5 [6.9] years). Over a mean (SD) observation time of 3.8 (2.3) years, with the longest observation at 7.5 years, FRAX was well calibrated in subgroups with negative VFA results. For individuals without a prior clinical fracture but with a positive VFA result, the 10-year FRAX-predicted MOF probability was 16.3% (95% CI, 15.7%-16.8%) without VFA information and 23.4% (95% CI, 22.7%-24.1%) with VFA information. The observed 10-year probabilities were 26.9% (95% CI, 26.0%-27.8%) and 11.2% (95% CI, 10.3%-12.1%), respectively, resulting in recalibration multipliers of 1.15 (95% CI, 0.87-1.43) for MOF and 1.31 (95% CI, 0.75-1.87) for hip fracture. For individuals with a prior clinical fracture and a positive VFA result, the 10-year FRAX-predicted probabilities were 25.0% (95% CI, 24.2%-25.7%) for MOF and 9.3% (95% CI, 8.7%-10.0%) for hip fracture. The observed 10-year probabilities were 38.1% (95% CI, 37.0%-39.1%) for MOF and 16.4% (95% CI, 15.4%-17.4%) for hip fracture, resulting in a recalibration multiplier of 1.53 (95% CI, 1.10-1.96) for MOF and 1.76 (95% CI, 1.17-2.35) for hip fracture. Good calibration (>0.90) was confirmed using the derived multipliers in the validation cohort.

**Conclusions and Relevance:**

Results of this prognostic study suggest that FRAX underestimated fracture risk in patients with VFA-identified fractures. Simple multipliers could recover FRAX calibration in individuals with VFA-identified fractures.

## Key Points

**Question:**

How accurate are FRAX-predicted fracture probabilities in the setting of a vertebral fracture identified on vertebral fracture assessment (VFA), and can they be improved with simple multipliers?

**Findings:**

In this prognostic study of more than 11 000 individuals, FRAX was well calibrated in subgroups without VFA-identified fractures but significantly underestimated major osteoporotic fractures (MOFs) and hip fracture risk in patients with VFA-identified fractures when VFA results were not used or the current method was used, which only allowed for a single prior fracture. Using multipliers for MOF and hip fracture restored calibration.

**Meaning:**

Findings of this study suggest that simple multipliers can recover FRAX calibration in individuals with VFA-identified fractures and substantially improve the agreement between observed and predicted fracture risk.

## Teaser

This prognostic study evaluates the calibration of a validated fracture risk prediction tool in a cohort of patients with a wide age range in Manitoba, Canada.

## Introduction

Vertebral fractures are prevalent in older individuals and have been identified in up to 18% in those 80 years or older, with an incidence rate of 10.7 per 1000 person-years in females and 5.7 per 1000 person-years in males older than 50 years.^1,2,3^ Vertebral fractures are a risk factor for subsequent clinical fractures, including hip fractures.^4,5^ However, only approximately 1 of 3 fractures are clinically diagnosed at the time of their occurrence.^6,7,8^

Vertebral fracture assessment (VFA) provides thoracolumbar lateral spine images using dual-energy x-ray absorptiometry (DXA) to detect vertebral fractures at the time of bone mineral density (BMD) measurement. Vertebral fracture assessment requires far less radiation exposure than standard lateral spine plain radiographs, with good sensitivity for detecting moderate to severe vertebral fractures.^9^ Prevalent vertebral fractures identified by VFA can predict future osteoporotic fractures even after adjusting for age, BMD, and prior clinical fractures.^10,11,12^ A VFA that is positive for fracture increases the risk for future incident hip, vertebral, and major osteoporotic fractures (MOFs) approximately 1.5- to 3-fold, with future fracture risk increasing with a higher number and grade of vertebral fractures detected on VFA after adjusting for baseline risk.^11,13,14^ Vertebral fracture assessment has been shown to be a cost-effective addition to fracture risk assessment and has been included in osteoporosis screening and management guidelines.^15,16,17,18^

FRAX is the most commonly used and widely validated fracture prediction tool worldwide, with tools available for more than 70 countries and in more than 31 languages.^19^ FRAX considers multiple clinical risk factors, including prior fracture. Prior fractures increase the hazard of future osteoporotic fractures by more than 80% and hip fractures by 35%.^20^ Vertebral fracture on DXA scan counts as a prior fracture in the FRAX calculation. However, FRAX does not distinguish between sites of prior fracture or between single and multiple fractures. Thus, currently, individuals with positive VFA results and prior clinical fracture would not affect the FRAX score. Moreover, vertebral fractures impart a greater risk for subsequent fractures than the average fracture risk used to model the outcome of prior fracture in FRAX.^21,22^ Therefore, FRAX would be expected to underestimate risk in individuals with a vertebral fracture detected on VFA, particularly in the presence of a prior clinical fracture. The objective of this study was to assess the calibration of FRAX and develop a simple method for improving FRAX-predicted fracture probability in the presence of VFA-identified vertebral fracture.

## Methods

### Data Sources

We performed a retrospective prognostic study using the Manitoba BMD Program’s DXA and VFA registry, which is linked to population-based administrative databases of the Canadian province of Manitoba. The University of Manitoba Health Research Ethics Board approved the study, and the Manitoba Health Information Privacy Committee approved data access and waived the requirement for signed consent in accordance with the Personal Health Information Act. We followed the Transparent Reporting of a Multivariable Prediction Model for Individual Prognosis or Diagnosis (TRIPOD) reporting guideline.^23^

The Manitoba BMD Program runs all clinical BMD testing and VFA in the province and maintains a database of all DXA scan results dating back to 1990 and all VFA results dating back to 2010. This population-based database has been shown to be nearly 100% complete and accurate.^24^

Residents of Manitoba are provided health services through a single public health care system.^25^ Information on health care visits, procedures, service types and their dates, and associated diagnosis codes were obtained from physician claims and hospital discharge databases via a unique personal health identification number, which can be used to link data within the various provincial databases. Physician billing claims use *International Classification of Diseases, Ninth Revision, Clinical Modification* (*ICD-9-CM*) codes, and hospital discharge abstracts use the *ICD-9-CM* prior to 2004 and *International Statistical Classification of Diseases and Related Health Problems, Tenth Revision, Canada* (*ICD-10-CA*) after 2004. Medication use was ascertained from the provincial pharmacy database, which records all medications dispensed in the outpatient setting.^26^ Deaths were ascertained from the Vital Statistic registry, which records all deaths that occur in Manitoba.^27^

### Study Population

We included all individuals who had a VFA recorded in the Manitoba BMD Registry between March 31, 2010, and March 31, 2018. We excluded those who were not registered for health care in Manitoba or those without coverage after DXA. Vertebral fracture assessment was added to the Manitoba BMD Program in 2010 if an individual met the following criteria: minimum T score of −1.5 or lower (lumbar spine, total hip, or femoral neck); age 70 years or older or age 50 to 69 years; and self-reported height loss of more than 5 cm, measured height loss of more than 2.5 cm, or at least 7.5 mg/d of prednisone at least 3 months in the preceding year as ascertained by the intake survey or direct assessment at the time of the DXA scan.

Using the modified algorithm-based qualitative (mABQ) approach, expert readers (including W.D.L.) coded VFAs as having a positive result (≥1 vertebral fractures detected) or negative result (0 vertebral fracture detected).^2^ The mABQ approach emphasizes end plate depression or cortical discontinuity or buckling as the defining feature of vertebral fracture and distinguishes this from nonfracture causes of vertebral deformity (eg, degenerative remodeling, Scheuermann disease, and Schmorl nodes). In a random sample of 127 images, of which one-half were reported to be positive for vertebral fracture and the other half to be negative for vertebral fracture, the interrater agreement between 2 expert readers, both of whom were blinded to the clinical readings, was high (κ scores of 0.86 and 0.78, respectively). The vertebral fractures identified on VFA images through the mABQ approach in the Manitoba BMD Program have been associated with incident fractures.^11^

### Incident Fracture Assessment

Incident fractures were ascertained from hospital discharge abstracts (*ICD-9-CM* or *ICD-10-CA*) and physician billing claims (*ICD-9-CM*) up to March 31, 2018, using previously validated fracture site–specific algorithms, which required site-specific reduction, fixation, or casting codes for hip and forearm fractures.^28,29^ Fracture date was defined as the first clinical encounter for the fracture. Codes of the same fracture type within 6 months of an incident fracture were considered to represent the same fracture. Fractures with high-trauma codes were excluded per previously published *ICD-9-CM* and *ICD-10-CA* criteria.^30^ Hip, clinical vertebral, forearm, and humerus fractures were collectively designated as MOFs.

### Fracture Probability Assessment

The 10-year probabilities of MOF and hip fracture were calculated for each individual using the country-specific (Canadian) FRAX tool (FRAX Desktop Multi-Patient Entry, version 3.8; Centre for Metabolic Bone Diseases, University of Sheffield).^31^ All FRAX calculations included BMD. Clinical risk factors included in the FRAX tool were collected as previously described.^32,33^ Briefly, height and weight were measured at the time of the DXA scan. Parental hip fracture, smoking status, high alcohol intake, rheumatoid arthritis, and secondary causes of osteoporosis were assessed from information collected directly from the patient-completed intake questionnaire at the time of each DXA scan. Prior clinical fracture was ascertained from population-based health care data in the linked provincial population-based health care databases using the same fracture case definitions as outlined under the incident fracture assessment. Prolonged glucocorticoid use was ascertained from the intake questionnaire and provincial pharmacy system and was defined as exposure for more than 3 months in the prior year.^26^ When considering VFA results, if at least 1 vertebral fracture was found on VFA, previous fracture was selected in FRAX. If a previous fracture was already selected in FRAX, no changes were made to the FRAX calculation.

### Statistical Analysis

Using a train-test approach, we randomly assigned the full cohort who underwent VFA using DXA to either the development cohort or the validation cohort. Baseline characteristics of the development and validation cohorts were compared using a 1-way analysis of variance for continuous variables and χ^2^ tests of independence for categorical variables. Two-sided *P* < .05 was considered to be statistically significant.

We estimated the cumulative incidence function (CIF) for MOF and hip fracture, taking into consideration the competing risk of death,^34^ stratified by prior non-VFA clinical fracture status and VFA results. The maximum observation time was 7.5 years, and the mean observation time was 3.8 years. Therefore, the CIF was linearly extrapolated out to 10 years. FRAX has demonstrated linearity in MOF and hip fracture prediction over the 10-year prediction period.^35^ The projected 10-year observed fracture probability was compared with FRAX-predicted 10-year fracture probability, with or without VFA results, in the development cohort. Moreover, we estimated recalibration multipliers for MOF and hip fracture prediction in individuals with or without a prior clinical fracture.

The recalibration multipliers were then applied to the FRAX predictions for the validation cohort. Calibration was reassessed by comparing the observed vs predicted fracture probabilities, stratified by prior clinical fracture status and VFA results. To measure the accuracy of the probabilistic predictions, we calculated a Brier score, rescaling each individual’s 10-year predicted probability of fracture for the actual observation time.^36^

Effect modification by age and sex were tested using the interaction terms age × VFA and sex × VFA. No effect modification was observed (*P* for interaction >.50), and therefore the analyses were not further stratified by age or sex. In individuals with a prior vertebral fracture, we did not know whether a subsequent positive VFA result corresponded with a prior or a new vertebral fracture. Thus, sensitivity analysis was conducted in which those with prior vertebral fracture were excluded. Statistical analyses were performed with Statistica, version 13.0 (StatSoft Inc) from August 7, 2022, to May 22, 2023.

## Results

The full cohort of 11 766 individuals was randomly assigned to the development cohort (n = 7854) or the validation cohort (n = 3912) (Table 1). The development cohort comprised 7349 females (93.6%) and 505 males (6.4%), with a mean (SD) age of 75.7 (6.8) years and a mean (SD) femoral neck T score of −2.1 (0.7). Of these patients, 2147 (27.3%) had a prior clinical fracture. The validation cohort consisted of 3713 females (94.9%) and 199 males (5.1%), with a mean (SD) age of 75.5 (6.9) years and a mean (SD) femoral neck T score of −2.0 (0.7). Among these patients, 1107 (28.3%) had a prior clinical fracture.

**Table 1.** Baseline Characteristics

Characteristic	Participants, No. (%)		*P* value
	Development (n = 7854)	Validation (n = 3912)	
Age, mean (SD), y	75.7 (6.8)	75.5 (6.9)	.15
Sex			
Female	7349 (93.6)	3713 (94.9)	.004
Male	505 (6.4)	199 (5.1)	
BMI, mean (SD)	26.2 (5.0)	26.3 (5.1)	.33
Prior clinical fracture	2147 (27.3)	1107 (28.3)	.27
Parental hip fracture	964 (12.3)	499 (12.8)	.46
Current smoker	646 (8.2)	316 (8.1)	.78
Prolonged glucocorticoid use	453 (5.8)	221 (5.6)	.79
Rheumatoid arthritis	368 (4.7)	153 (3.9)	.054
Secondary causes of osteoporosis	1267 (16.1)	662 (16.9)	.28
High alcohol intake	17 (0.2)	11 (0.3)	.497
Femoral neck T score, mean (SD)	−2.1 (0.7)	−2.0 (0.7)	.12
FRAX-predicted MOF before VFA, mean (SD), %	17.0 (8.2)	16.9 (8.4)	.69
FRAX-predicted hip fracture before VFA, mean (SD), %	5.9 (5.9)	5.8 (6.1)	.49
Positive VFA result	1287 (16.4)	631 (16.1)	.72

Abbreviations: BMI, body mass index (calculated as weight in kilograms divided by height in meters squared); MOF, major osteoporotic fracture; VFA, vertebral fracture assessment.

Before including the results of VFA, the mean (SD) 10-year FRAX probabilities of fracture were similar between the development cohort (MOF: 17.0% [8.2%]; hip fracture: 5.9% [5.9%]) and validation cohort (MOF: 16.9% [8.4%]; hip fracture: 5.8% [6.1%]). There was a positive VFA result in 1287 individuals (16.4%) in the development cohort and 631 individuals (16.1%) in the validation cohort. Baseline characteristics were balanced between the 2 cohorts except the validation cohort had a slightly higher percentage of women (94.9% vs 93.6%; *P* = .004) (Table 1).

The mean (SD) observation time in both cohorts was 3.8 (2.3) years, with the longest observation being 7.5 years (eTable in Supplement 1). Incident MOF occurred in 598 individuals (7.6%) in the development cohort, of which 222 fractures (2.8%) involved the hip, and in 328 individuals (8.4%) from the validation cohort, of which 116 fractures (3.0%) involved the hip.

**Figure 1.**
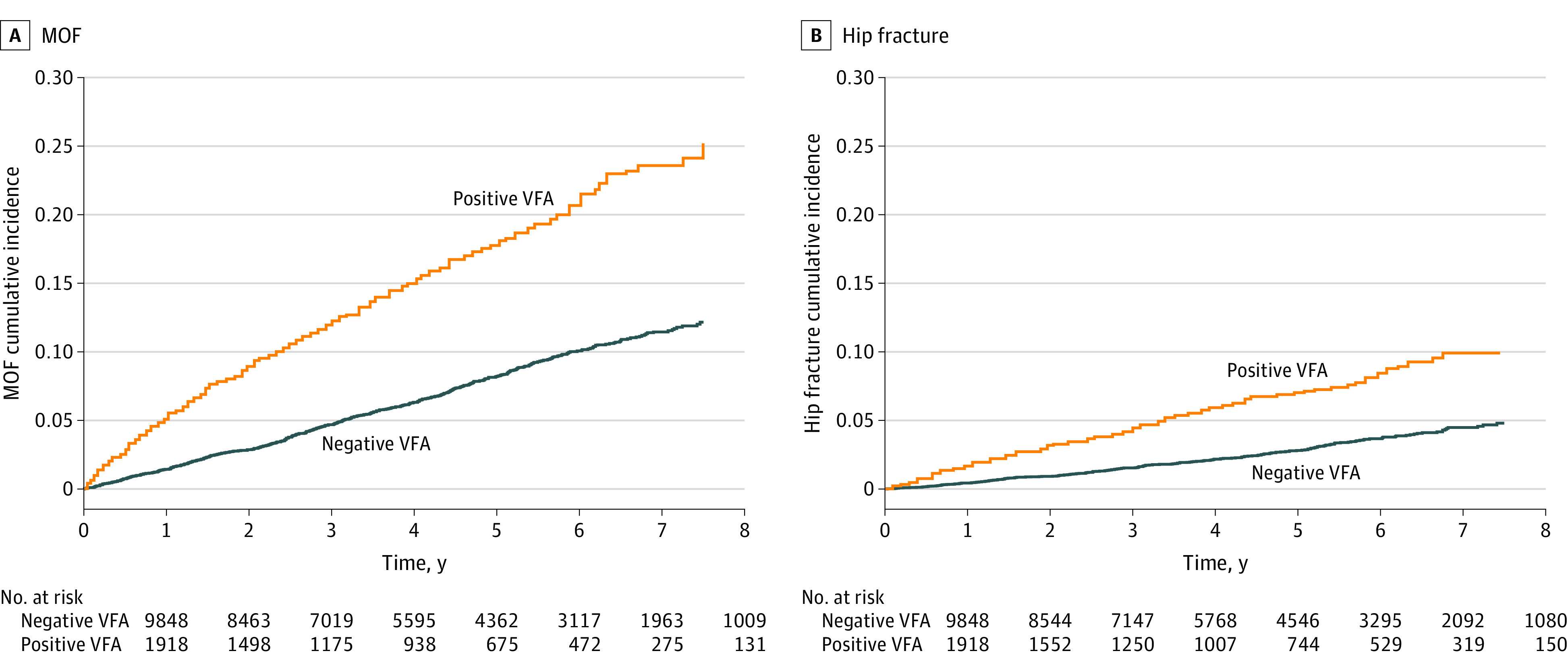
Major Osteoporotic Fracture (MOF) and Hip Fracture Cumulative Incidence Function by Vertebral Fracture Assessment (VFA) Status Positive VFA indicates that 1 or more fractures were detected, whereas negative VFA indicates that no vertebral fracture was detected.

**Figure 2.**
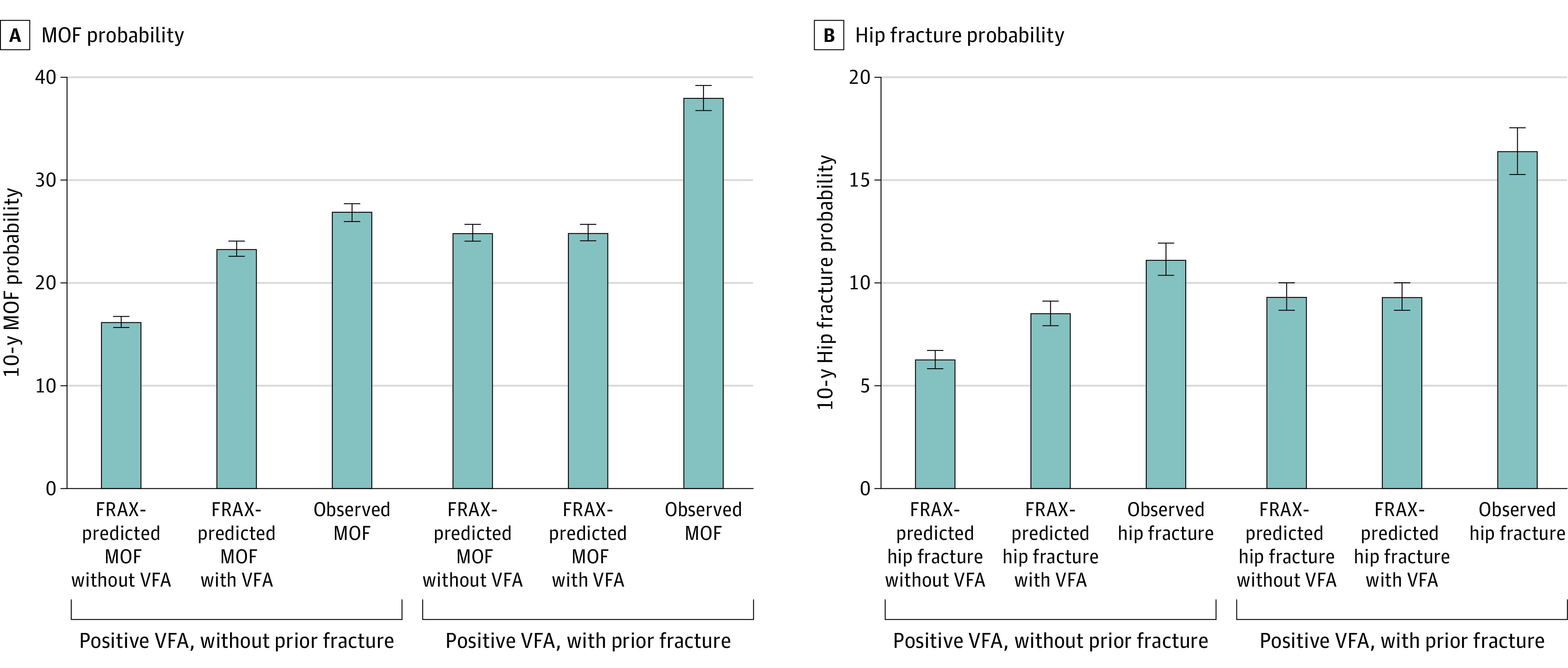
FRAX-Predicted vs Observed 10-Year Major Osteoporotic Fracture (MOF) and Hip Fracture Probability in Patients With Positive Vertebral Fracture Assessment (VFA) Result in the Development Cohort Positive VFA indicates that 1 or more fractures were detected, whereas negative VFA indicates that no vertebral fracture was detected. Error bars represent 95% CIs.

The CIF of MOF and hip fracture by VFA status followed near-linear curves (Figure 1). For those in the development cohort without a prior clinical fracture but with a positive VFA result, the 10-year FRAX-predicted MOF probability was 16.3% (95% CI, 15.7%-16.8%) without VFA information and 23.4% (95% CI, 22.7%-24.1%) with VFA information; the observed 10-year CIF was 26.9% (95% CI, 26.0%-27.8%), resulting in a recalibration multiplier of 1.15 (95% CI, 0.87-1.43) for MOF in an individual without prior clinical fracture (Figure 2A). For those in the development cohort with a prior clinical fracture and a positive VFA result, the 10-year FRAX-predicted MOF probability with and without VFA information was 25.0% (95% CI, 24.2%-25.7%) for both groups since a second fracture detected by VFA does not change the FRAX output. The observed 10-year MOF probability was 38.1% (95% CI, 37.0%-39.1%), resulting in a recalibration multiplier of 1.53 (95% CI, 1.10-1.96) for MOF in an individual with a prior clinical fracture.

In patients without prior clinical fracture but a positive VFA result, the 10-year FRAX-predicted hip fracture probability was 6.3% (95% CI, 5.8%-6.7%) without VFA information and 8.5% (95% CI, 7.9%-9.1%) with VFA information, and the observed 10-year CIF was 11.2% (95% CI, 10.3%-12.1%), resulting in a recalibration multiplier of 1.31 (95% CI, 0.75-1.87) for hip fracture in an individual without prior clinical fracture (Figure 2B). In those with a prior clinical fracture and positive VFA result, the 10-year FRAX-predicted hip fracture probability with and without VFA information was 9.3% (95% CI, 8.7%-10.0%) for both groups. The observed 10-year hip fracture probability was 16.4% (95% CI, 15.4%-17.4%), resulting in a recalibration multiplier of 1.76 (95% CI, 1.17-2.35) for hip fracture in an individual with a prior clinical fracture.

After excluding those with a prior vertebral fracture diagnosis (178 in the development cohort; 82 in the validation cohort), 1109 individuals (86.2%) with a positive VFA result remained in the development cohort, and 549 individuals (87.0%) with a positive VFA result remained in the validation cohort. The recalibration multipliers for these individuals were similar in this sensitivity analysis: 1.15 (95% CI, 0.87-1.43) for MOF and 1.31 (95% CI, 0.75-1.87) for hip fracture in individuals without prior clinical fracture and 1.46 (95% CI, 0.94-1.98) for MOF and 1.70 (95% CI, 1.04-2.36) for hip fracture in individuals with a prior fracture (other than vertebral fracture).

**Figure 3.**
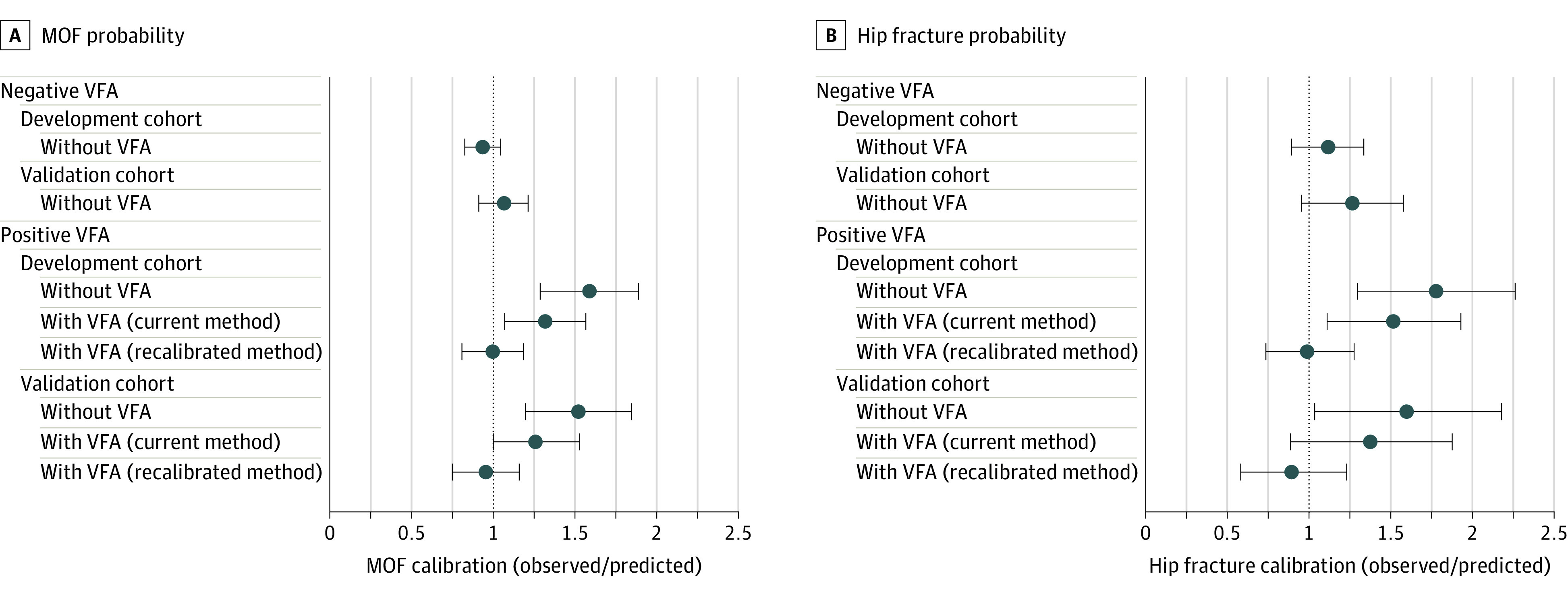
Calibration Between Observed and FRAX-Predicted Major Osteoporotic Fracture (MOF) and Hip Fracture Probability Positive vertebral fracture assessment (VFA) indicates that 1 or more fractures were detected, whereas negative VFA indicates that no vertebral fracture was detected. Error bars represent 95% CIs.

When the recalibration multipliers were applied to the FRAX-predicted MOF and hip fracture probabilities in the validation cohort, the observed calibration ratios were 0.96 (95% CI, 0.76-1.16) and 0.90 (95% CI, 0.58-1.22), respectively (Figure 3). Brier scores were low for incident MOF prediction (0.002 in the development cohort; 0.016 in the validation cohort) and hip fracture prediction (0.003 in the development cohort; 0.008 in the validation cohort) in individuals with a negative VFA result (Table 2). In those from the validation cohort with a positive VFA result, Brier scores decreased (improved) with the addition of VFA data from 0.160 to 0.132 for MOF prediction and 0.052 to 0.043 for hip fracture prediction. There was further reduction (improvement) in Brier scores after recalibration multipliers were incorporated into the predictions (0.074 for MOF prediction and 0.008 for hip fracture prediction).

**Table 2.** Brier Scores for Incident Fracture Prediction by Vertebral Fracture Assessment (VFA) Status in Development and Validation Cohorts

VFA status	Predicted MOF, Brier scores			Predicted hip fracture, Brier scores		
	Without VFA	With VFA	With recalibration	Without VFA	With VFA	With recalibration
**Development cohort**						
Negative VFA results	0.002	0.002	0.002	0.003	0.003	0.003
Positive VFA results	0.131	0.102	0.047	0.056	0.047	0.015
**Validation cohort**						
Negative VFA results	0.016	0.016	0.016	0.008	0.008	0.008
Positive VFA results	0.160	0.132	0.074	0.052	0.043	0.008

Abbreviation: MOF, major osteoporotic fracture.

## Discussion

Results of this prognostic study confirmed that a vertebral fracture detected on VFA identified a situation in which FRAX significantly underestimated fracture risk. This outcome was exaggerated for individuals with a prior non–VFA-detected clinical fracture since the previous fracture option was already selected and a positive VFA result was not considered in FRAX. In these individuals, FRAX underestimated MOF risk by 53% (recalibration multiplier: 1.53) and hip fracture risk by 76% (recalibration multiplier: 1.76). Although less marked in individuals without a prior clinical fracture, in which a positive VFA result will prompt the previous fracture option to be selected, FRAX still underestimated risk in these individuals, likely due to vertebral fractures conveying higher subsequent fracture risk than any other type of fracture.^37^ However, simple recalibration multipliers can recover FRAX calibration in individuals with positive VFA results.

FRAX has proven to be robust across many populations and remains the best validated tool for fracture risk prediction.^19^ Other factors that are not currently considered in FRAX have been associated with miscalibration, including recency of fracture, type 2 diabetes, multiple sclerosis, hip axis length, exposure to higher-than-average doses of glucocorticoids, discordant lumbar spine BMD, multiple falls, trabecular bone score, and chronic kidney disease, and have led to the planning of the next iteration of FRAX.^33,38,39,40^ Integration of VFA results as a FRAX input separate from prior clinical fracture may be a consideration for future updates of FRAX. Meanwhile, this can be accommodated among the growing list of posttranslational adjustments that will be available through the FRAXplus website.

### Strengths and Limitations

Strengths of this study include use of the Manitoba BMD Registry, which contains one of the largest VFA data sets worldwide for a cohort that is representative of routine clinical practice. Additionally, VFA interpretation was performed by a small group of highly experienced readers of DXA scan and VFA results, all of whom have been certified by the International Society for Clinical Densitometry. The study also used stringent fracture definitions that have been validated against x-ray review.^29^

The study has several limitations. The recalibration multipliers had wide CIs, reflecting a degree of imprecision that would be attenuated by a larger VFA cohort with associated fracture outcomes, a study of which has not been published. Although the recalibration multipliers derived from the present cohort were internally validated, they have not been externally validated. These findings are similar to those previously reported in a study of 2852 females aged 75 to 80 years, which found that a VFA-identified vertebral fracture increased the risk of subsequent MOF by approximately 20% to 40% and hip fracture by approximately 50% to 70%, providing some degree of external validation.^13^ Further external validation is provided by the Reykjavik Study fracture register, which found that FRAX-based fracture probabilities needed to be increased by approximately 10%, 20%, and 30% in individuals with a history of 2, 3, and 4 or more prior fractures, respectively.^38^ These numbers were lower than those seen in this study, likely due to vertebral fracture conveying higher subsequent fracture risk than other fractures.

Although this cohort included males and females with a large age range, the VFA criteria restricted the cohort to specific parameters and thus was composed primarily of older females, reflecting the clinical population typically referred for DXA scans. The results may not be generalizable to males or other subgroups who were not well represented in the cohort. The prevalence of vertebral fractures identified on VFA in this study was within the range reported in other studies, which varies widely depending on the risk profile of the cohort undergoing VFA.^13,41,42^

The mean follow-up time was 3.8 years, with a maximum follow-up of 7.5 years. Thus, the observed 10-year fracture risk was extrapolated beyond 7.5 years. While longer follow-up duration would increase the validity of the estimated observed 10-year fracture risk, linear extrapolation was justified by the linearity of the MOF and hip fracture CIF curves and previously demonstrated linear agreement between observed and FRAX-predicted fracture risk over 10 years.^35^ No effect modification by age or sex was found in this study, but it was likely underpowered to detect such differences given the small proportion of males and individuals younger than 70 years in the clinical cohort.

## Conclusions

In this prognostic study of individuals who underwent VFA, FRAX underestimated the fracture risk in patients with vertebral fracture identified on VFA even when prior fracture was included in the calculation. Simple recalibration multipliers for a positive VFA result were able to substantially improve the agreement between observed and predicted fracture risk.

## Notes

Supplement 1. **eTable.** Incident Fracture Outcomes

Supplement 2.
Data Sharing Statement
