# Supplementary material for: Adjusting FRAX Estimates of Fracture Probability Based on a Positive Vertebral Fracture Assessment
Source: JAMA Netw Open. 2023 Aug 17;6(8):e2329253. doi: 10.1001/jamanetworkopen.2023.29253 (PMC10436131; doi:10.1001/jamanetworkopen.2023.29253)
Supplement: Supplement 1. — eTable. Incident Fracture Outcomes [file jamanetwopen-e2329253-s001.pdf]

Supplemental Online Content

Ye C, Leslie WD, Morin SN, et al. Adjusting FRAX estimates of fracture probability based on a positive vertebral fracture assessment. *JAMA Netw Open*. 2023;6(8):e2329253. doi:10.1001/jamanetworkopen.2023.29253

eTable. Incident Fracture Outcomes

This supplemental material has been provided by the authors to give readers additional information about their work.

© 2023 Ye C et al. *JAMA Network Open.*

eTable. Incident Fracture Outcomes

	Development cohort N=7854 n (%)	Validation cohort N=3912 n (%)	p-value
Mean observation time, mean (SD), (years)	3.8 (± 2.3)	3.8 (± 2.3)	0.864
Incident MOF	598 (7.6)	328 (8.4)	0.144
Incident hip fracture	222 (2.8)	116 (3.0)	0.671
Incident humerus fracture	128 (1.6)	74 (1.9)	0.303
Incident forearm fracture	149 (1.9)	80 (2.0)	0.584
Incident vertebral fracture	143 (1.8)	86 (2.2)	0.162

MOF=major osteoporotic fracture. All values expressed as mean ± SD or count (%).

© 2023 Ye C et al. *JAMA Network Open.*
