# Supplementary material for: Adjusting FRAX Estimates of Fracture Probability Based on a Positive Vertebral Fracture Assessment
Source: JAMA Netw Open. 2023 Aug 17;6(8):e2329253. doi: 10.1001/jamanetworkopen.2023.29253 (PMC10436131; doi:10.1001/jamanetworkopen.2023.29253)
Supplement: Supplement 2. — Data Sharing Statement [file jamanetwopen-e2329253-s002.pdf]

Data Sharing Statement

Ye. Adjusting FRAX Estimates of Fracture Probability Based on a Positive Vertebral Fracture Assessment. *JAMA Netw Open*. Published August 17, 2023.
doi:10.1001/jamanetworkopen.2023.29253

Data
Data available: No
